# Active cancer as the main predictor of mortality for COVID-19 in oncology patients in a specialized center

**DOI:** 10.3389/pore.2023.1611236

**Published:** 2023-09-07

**Authors:** Freddy Villanueva-Cotrina, Juan Velarde, Ricardo Rodriguez, Alejandra Bonilla, Marco Laura, Tania Saavedra, Diana Portillo-Alvarez, Yovel Bustamante, Cesar Fernandez, Marco Galvez-Nino

**Affiliations:** ^1^ Department of Pathology, Instituto Nacional de Enfermedades Neoplasicas, Lima, Peru; ^2^ Academic Department of Medical Microbiology, Universidad Nacional Mayor de San Marcos, Lima, Peru; ^3^ Department of Infectious Diseases, Instituto Nacional de Enfermedades Neoplasicas, Lima, Peru; ^4^ Academic Department of Medical Technologist, Universidad Nacional Mayor de San Marcos, Lima, Peru; ^5^ Department of Radiodiagnosis, Instituto Nacional de Enfermedades Neoplasicas, Lima, Peru; ^6^ Department of Critical Care Medicine, Instituto Nacional de Enfermedades Neoplasicas, Lima, Peru; ^7^ Professional School of Human Medicine, Universidad Privada San Juan Bautista, Lima, Peru; ^8^ Professional School of Human Medicine, Universidad de Piura, Lima, Peru; ^9^ Department of Medical Oncology, Instituto Nacional de Enfermedades Neoplasicas, Lima, Peru

**Keywords:** SARS-CoV-2, imaging study, biomarkers, death prognosis, active cancer

## Abstract

**Introduction:** The role of the type, stage and status of cancer in the outcome of COVID-19 remains unclear. Moreover, the characteristic pathological changes of severe COVID-19 reveled by laboratory and radiological findings are similar to those due to the development of cancer itself and antineoplastic therapies.

**Objective:** To identify potential predictors of mortality of COVID-19 in cancer patients.

**Materials and methods:** A retrospective and cross-sectional study was carried out in patients with clinical suspicion of COVID-19 who were confirmed for COVID-19 diagnosis by RT-PCR testing at the National Institute of Neoplastic Diseases between April and December 2020. Demographic, clinical, laboratory and radiological data were analyzed. Statistical analyses included area under the curve and univariate and multivariate logistic regression analyses.

**Results:** A total of 226 patients had clinical suspicion of COVID-19, the diagnosis was confirmed in 177 (78.3%), and 70/177 (39.5%) died. Age, active cancer, leukocyte count ≥12.8 × 109/L, urea ≥7.4 mmol/L, ferritin ≥1,640, lactate ≥2.0 mmol/L, and lung involvement ≥35% were found to be independent predictors of COVID-19 mortality.

**Conclusion:** Active cancer represents the main prognosis factor of death, while the role of cancer stage and type is unclear. Chest CT is a useful tool in the prognosis of death from COVID-19 in cancer patients. It is a challenge to establish the prognostic utility of laboratory markers as their altered values it could have either oncological or pandemic origins.

## Introduction

COVID-19 disease leads to severe pneumonia, metabolic acidosis, coagulation dysfunction, multiple organ failure, and eventually, death [1–4]. Cancer patients usually have a compromised immune system with a higher risk of death in COVID-19 compared to those without cancer [5–8], but it is not clear if the type, stage and status of cancer play a role in the outcome of the disease.

COVID-19 diagnosis is based on clinical evaluation and confirmed by the detection of viral RNA in respiratory samples [9]. Furthermore, certain blood laboratory parameters and chest computed tomography (CT) findings could reveal characteristic pathological changes and the clinical course of COVID-19 in oncology patients. Altered levels of C-reactive protein (CRP), neutrophil and lymphocyte counts, ferritin, D-dimer, and lactate dehydrogenase [10–12], as well as a high CO-RADS score and the presence of some abnormal chest CT findings have been associated with the presentation of severe complications of COVID-19 [13, 14].

However, malignant neoplasms lead to similar laboratory and radiological alteration findings due to pathological events in the development of cancer itself (acute renal failure, disseminated intravascular coagulation, impaired cellular immunity, organ failure, respiratory failure) [15–21] and antineoplastic therapies (immunosuppression, hepatotoxicity) [22, 23]. Therefore, it is unknown whether these potential predictors are useful for detecting the clinical course of the disease in cancer patients.

We aimed to identify predictors of mortality of COVID-19 in cancer patients by the joint study of the clinical characteristics, laboratory and radiological findings, and their association with a higher risk of a fatal outcome.

## Materials and methods

### Study design and population

This retrospective and cross-sectional study was carried out in patients hospitalized at the Instituto Nacional de Enfermedades Neoplasicas (INEN) in Lima—Peru between April and December 2020. The study sample included patients who presented with a clinical suspicion of COVID-19 and were confirmed for COVID-19 diagnosis by RT-PCR testing. Patients with a clinical or radiological diagnosis of COVID-19 without a positive RT-PCR test were excluded. When patients had two positive results for COVID-19 by RT-PCR, only the first one was considered.

### Clinical, laboratory, radiological and outcome data

Demographic, clinical, laboratory, radiological and outcomes data were collected blind from the medical records and the INEN informatic system. Blood sample tests and chest CT scans were performed within 24 h after sampling for the COVID-19 molecular diagnosis. While clinical outcome, mortality or survival, was considered until 30 days after the molecular study was performed. A specialist physician from the Department of Infectious Diseases evaluated the records of the clinical presentation, while two specialist physicians from the Department of Medical Oncology evaluated the stage and status of cancer in the patients. Two specialist physicians from the Department of Radiodiagnosis evaluated the chest CT images to determine the presence of abnormal findings, the percentage of affected lung, and the COVID-19 Reporting and Data System (CO-RADS) score classification system.

To determine the percentage of affected lung, each of the five lung lobes was scored visually on a scale from 0 to 5, (where 0 indicates no involvement; 1, less than 5%; 2, 5%–25%; 3, 26%–49%; 4, 50%–75%; and 5, more than 75%). The total CT score was the sum of the individual lobar scores and ranged from 0 (no involvement) to 25 (maximum involvement) [24]. The CO-RADS system assessed lung damage on a chest CT to predict the likelihood of COVID-19 pneumonia using a scale from 1 (very low) to 5 (very high). A high CO-RADS score indicates a high probability of COVID-19; thus, the grouped frequency of COVID-19 in categories 1, 2, 3, 4 and 5 corresponds to 8.8%, 11.1%, 24.6%, 61.9% and 89.6% involvement, respectively) [25].

### Definitions

Cases with clinical suspicion of COVID-19: Patients present any of the following symptoms: fever, cough, fatigue, headache, dyspnea, myalgia, diarrhea, tachypnea, chest pain, anosmia, and ageusia.

Confirmed cases of COVID-19: Patients who tested positive for COVID-19 in the molecular study (PCR-RT) from nasopharyngeal swab samples.

Active cancer: Presence of cancer progression or recurrence after treatment.

Advanced cancer: Presence of distant metastatic disease.

Clinical scenario: Clinical presentation of patients according to WHO [26].

### Compliance with ethics guidelines

All the cases in this study were part of the medical care routine of the INEN. No informed consent from any patient was obtained since this study used laboratory test registers, radiological reports of the INEN informatic system, and patient medical records in obtaining data which were used protecting the identity of the patients. The protocol was presented to the Research Committee of the INEN and approved for its implementation with designated protocol number INEN 20-49.

### Statistical analysis

Categorical variables were analyzed using the chi-squared or Fisher’s exact test, as appropriate, and presented as frequencies and percentages. Quantitative variables were described as means and standard deviation or medians and interquartile ranges, depending on the distribution of the data. Quantitative variables were evaluated using the Student’s t-test when there was a normal distribution of the data and, when not, using the Mann‐Whitney *U* test. Receiver operating characteristic (ROC) curve analysis provided the sensitivity and specificity of the laboratory tests and radiological findings for the prediction of mortality from COVID-19. In addition, it allowed us to obtain the best cut-off points for the categorization of these variables according to the Youden Index. Finally, the univariate and multivariate regression analyses were performed to obtain the odds ratio (OR) as a measure of association between the variables and the mortality prediction of COVID-19, considering only the associated variables in the bivariate analysis for building the final multivariate model. For all analyses, statistical significance was set at *p* < 0.05. All analyses were performed using Stata version 14.0.

## Results

During the study period, 226 patients had clinical suspicion of COVID-19 from which 177 (78.3%) were confirmed. Of the 177 positive cases of COVID-19, most patients were between 16 and 59 years old (84, 47.5%), male (96, 54.2%), had solid tumors (95, 53.7%), non-advanced stage (104, 58.8%) and active status cancer (111, 62.7%). Of these, 70 (39.5%) died, one-third of the deaths of patients with COVID-19 occurred within the first 5 days (24/70, 34.4%), and the vast majority (52/70, 74.3%) had died by day 15 after molecular study sampling**.**

### Prediction of COVID-19 mortality

The deceased patients were older (62.5 vs. 42.0; *p* ≤ 0.001), had severe-critical clinical scenario (56.6% vs. 26.7%; *p* ≤ 0.001) and active cancer (48.7% vs. 24.2%; *p* = 0.001) (Table 1).

**TABLE 1:** Demographic, clinical and oncological characteristics of 177 cases of cancer patients with COVID-19 according to mortality.

Variables		Survivors **N (%)**	Dead **N (%)**	*p*-value
Age groups				**<0.001**
	0–15	15 (83.3)	3 (16.7)	
	16–59	60 (71.4)	24 (28.6)	
	≥60	32 (42.7)	43 (57.3)	
Age (years)	Median ± Interquartile range	48 (28–60)	62.5 (50–72)	**<0.001**
Gender				0.360
	Female	46 (56.8)	35 (43.2)	
	Male	61 (63.5)	35 (36.5)	
Comorbidity				0.328
	No	66 (63.5)	38 (36.5)	
	Yes	41 (56.2)	32 (43.8)	
Diabetes				0.501
	No	98 (59.8)	66 (40.2)	
	Yes	9 (69.2)	4 (30.8)	
Arterial hypertension				0.321
	No	93 (62.0)	57 (38.0)	
	Yes	14 (51.9)	13 (48.1)	
Lung disease				0.934
	No	99 (60.4)	65 (39.6)	
	Yes	8 (61.5)	5 (38.5)	
Obesity				0.443
	No	101 (61.2)	64 (38.8)	
	Yes	6 (50.0)	6 (50.0)	
Other comorbidities				0.886
	No	79 (60.8)	51 (39.2)	
	Yes	28 (59.6)	19 (40.4)	
Clinical scenario				**<0.001**
	Mild/moderate	74 (73.3)	27 (26.7)	
	Severe/critical	33 (43.4)	43 (56.6)	
Type of cancer				0.172
	Hematological malignancies	54 (65.9)	28 (34.1)	
	Solid tumors	53 (55.8)	42 (44.2)	
Oncological diagnosis				0.208
	Acute lymphocytic leukemia	16 (72.7)	6 (27.3)	
	Non-Hodgkin lymphoma	21 (65.6)	11 (34.4)	
	^a^Other hematologic malignancies	17 (60.7)	11 (39.3)	
	Urological cancer	9 (47.4)	10 (52.6)	
	Breast cancer	10 (41.7)	14 (58.3)	
	^b^Other solid tumors	34 (65.4)	18 (34.6)	
Cancer stage				0.082
	Non-advanced	68 (65.4)	36 (34.6)	
	Advanced	36 (52.2)	33 (47.8)	
Cancer status				**0.001**
	Non-active	50 (75.8)	16 (24.2)	
	Active	57 (51.3)	54 (48.7)	

^a^ Acute myeloid leukemia, chronic myeloid leukemia, mixed phenotype leukemia, multiple myeloma.

^b^ Cervix cancer, head and neck cancer, gastrointestinal cancer, lung cancer, liver and bile duct cancer, thyroid cancer, sarcoma, skin cancer, and other cancer.

Bold format means statistical significance of the *p* value.

Likewise, these patients had a higher leukocyte count (10.4×10^9^/L vs. 6.6×10^9^/L; *p* = 0.001), neutrophil count (8.63 × 10^9^/L vs. 5.26 × 10^9^/L; *p* = 0.001), RNL (15.1 vs. 7.8; *p* = 0.001), D-dimer (2,136 ng/mL vs. 1,034 ng/mL; *p* = 0.002), LDH (329 U/L vs. 249 U/L; *p* = 0.004), urea (6.0 mmol/L vs. 4.0 mmol/L; *p* ≤ 0.001), creatinine (56 umol/L vs. 48 umol/L; *p* = 0.018, lactate (1.9 mmol/L vs. 1.3 mmol/L; *p* ≤ 0.001), lung involvement (40% vs. 20%; *p* ≤ 0.001) and crazy paving (56.3% vs. 32.3%) (Table 2).

**TABLE 2:** Comparison of laboratory tests and CT findings in 177 cancer patients with COVID-19 according to mortality.

Variables	Survivors	Dead	*p*-value
	**N (%)**	**N (%)**	
C-reactive protein mg/L	105.85 (39.00–173.90)	122.85 (74.30–198.75)	0.115
Leukocytes ×10^9^/L	6.65 (3.64–11.4)	10.40 (7.00–17.80)	**0.001**
Neutrophils ×10^9^/L	5.26 (3.25–10.03)	8.63 (6.37–14.58)	**0.001**
Neutrophil/lymphocyte ratio	7.80 (3.40–15.20)	15.11 (6.00–24.18)	**0.001**
Platelets ×10^9^/L	222.00 (145.00–326.00)	199.50 (94.00–326.00)	0.225
Fibrinogen g/L	5.75 ± 2.11	5.84 ± 2.31	0.802
D-dimer ng/mL	1,034.00 (644.00–2,925.00)	2,136.50 (948.00–7,989.00)	**0.002**
Lactate dehydrogenase U/L	249.00 (186.00–362.00)	329.00 (235.00–482.00)	**0.004**
Urea mmol/L	4.30 (3.10–5.10)	6.00 (4.85–9.30)	**<0.001**
Creatinine umol/L	48.00 (37.00–62.00)	56.00 (39.00–83.00)	**0.018**
Ferritin ng/mL	679.0 (327.00–1,180.00)	912.00 (371.50–1895.00)	0.070
Oxygen saturation %	95.80 (92.90–97.40)	95.1 (91.4–96.8)	0.273
Lactate mmol/L	1.30 (1.00–1.80)	1.90 (1.30–2.80)	**<0.001**
Lung involvement %	20 (10–40)	40 (20–70)	**<0.001**
Score CO-RADS			0.436
CO-RADS 1	6 (60.0)	4 (40.0)	
CO-RADS 2	12 (63.2)	7 (36.8)	
CO-RADS 3	10 (62.5)	6 (37.5)	
CO-RADS 4	21 (61.8)	13 (38.2)	
CO-RADS 5	44 (54.3)	37 (45.7)	
Consolidation			0.577
No	43 (60.6)	28 (39.4)	
Yes	50 (56.2)	39 (43.8)	
Nodular pattern			0.722
No	77 (58.8)	54 (41.2)	
Yes	16 (55.2)	13 (44.8)	
Frosted glass			0.983
No	11 (57.9)	8 (42.1)	
Yes	82 (58.2)	59 (41.8)	
Crazy paving			**0.003**
No	65 (67.7)	31 (32.3)	
Yes	28 (43.7)	36 (56.3)	
Organizing pneumonia			0.397
No	84 (57.1)	63 (42.9)	
Yes	9 (69.2)	4 (30.8)	
Vessel thickening			0.090
No	37 (67.3)	18 (32.7)	
Yes	56 (53.3)	49 (46.7)	

Bold format means statistical significance of the *p* value.

The evaluation of the area under the curve showed that urea ≥7.4 mmol/L had the highest ability to predict death, with AUC 0.751 (S = 40.63% and E = 91.75%), followed by lactate ≥2.0 mmol/L, AUC 0.686 (S = 47.54% and E = 86.49%) and lung involvement ≥35%, AUC 0.662 (S = 59.70%, E = 66.67%) (Table 3).

**TABLE 3:** Prognostic capacity of death of laboratory tests and CT findings in cancer patients with COVID-19.

Variables	Reference values	AUC	95% CI	Cut-off	Se (%)	Sp (%)	Youden index
C-reactive protein mg/L	0–5	0.576	0.484–0.669	≥189.3	32.14	80.61	12.75
Leukocytes ×10^9^/L	4.68–11.8	0.654	0.571–0.737	≥12.8	37.14	83.18	20.32
Neutrophils ×10^9^/L	1.6–7.0	0.656	0.571–0.741	≥7.51	68.18	63.27	31.45
Neutrophil/lymphocyte ratio	NA	0.655	0.570–0.741	≥14.5	54.55	71.43	25.98
Platelets ×10^9^/L	182–393	0.446	0.357–0.535	≥393	14.29	87.85	2.14
Fibrinogen g/L	2.0–4.0	0.492	0.393–0.591	≥9.39	10.53	98.86	9.39
D-dimer ng/mL	<270	0.645	0.557–0.734	≥1,345	66.13	62.38	28.51
Lactate dehydrogenase U/L	120–246	0.633	0.548–0.717	≥329	50.75	71.57	22.32
Urea mmol/L	2.5–7.1	**0.751**	0.673–0.830	≥7.4	40.63	91.75	32.38
Creatinine umol/L	46–110	0.608	0.518–0.698	≥71.00	35.82	87.13	22.95
Ferritin ng/mL	17.9–464	0.603	0.492–0.713	≥1,640	31.82	87.69	19.51
Oxygen saturation %	95–100	0.445	0.347–0.543	≥86.9	93.44	8.00	1.44
Lactate mmol/L	<2.0	0.686	0.596–0.777	≥2.0	47.54	86.49	34.03
Lung involvement %	NA	0.662	0.577–0.747	≥35	59.70	66.67	26.37
CO-RADS score	NA	0.540	0.455–0.624	≥5	55.22	52.69	7.91

AUC, area under the curve; CI, confidence interval; Se, sensibility; Sp, specificity; NA, Not applicable.

Bold format means statistical significance of the *p* value.

Finally, in the bivariate regression analysis, a higher probability of death was found in patients over 60 years of age (OR 6.72; *p* = 0.005) compared to those under 16 years of age and with increasing age per year (OR 1.04; *p* ≤ 0.001), severe-critical clinical scenario (OR 3.57; *p* ≤ 0.001), active cancer (OR 2.96; *p* = 0.002), advanced cancer (OR 1.61; *p* = 0.082), leukocyte count ≥12.8 × 10^9^/L (OR 2.92; *p* = 0.003), neutrophil count ≥7.51 × 10^9^/L (OR 3.55; *p* ≤ 0.001), RNL ≥14.5 (OR 3.00; *p* = 0.001), fibrinogen ≥9.39 g/L (OR 9.94; *p* = 0.035), D-dimer ≥1,345 ng/mL (OR 2.57; *p* = 0.003), LDH ≥329 U/L (OR 2.54; *p* = 0.004), urea ≥7.4 mmol/L (OR 7.31; *p* ≤ 0.001), creatinine ≥71 umol/L (OR 3.77; *p* = 0.001), ferritin ≥1,640 (OR 3.09; *p* = 0.017), lactate ≥2.0 mmol/L (OR 6.86; *p* ≤ 0.001), lung involvement ≥35% (OR 3.27; *p* ≤ 0.001), crazy paving (OR 2.58; *p* = 0.001) and vessel thickening (OR 1.91; *p* = 0.011), and a lower probability in patients with oxygen saturation ≥86.9% (OR 0.45; *p* = 0.029). Multivariate analysis found an association with a higher probability of death with increasing age per year (OR 1.04; *p* = 0.001), active cancer (OR 7.56; *p*≤0.001), leukocyte count ≥12.8 × 10^9^/L (OR 3.00; *p* = 0.022), urea ≥7.4 mmol/L (OR 3.20; *p* = 0.034), ferritin ≥1,640 (OR 7.22; *p* = 0.005), lactate ≥2.0 mmol/L (OR 4.79; *p* = 0.002) and lung involvement ≥35% (OR 4.34; *p* = 0.002) (Table 4).

**TABLE 4:** Factors associated with mortality in cancer patients with COVID-19.

Variables	Raw model			Adjusted model		
	OR	95% CI	*p*-value	OR	95% CI	*p*-value
Age groups						
0–15	Ref.					
16–59	2.00	0.53–7.54	0.306			
≥60	6.72	1.79–25.19	0.005			
Age (years)	**1.04**	**1.02–1.06**	**<0.001**	**1.04**	**1.02–1.07**	**0.001**
Gender						
Male	Ref.					
Female	0.75	0.41–1.38	0.361			
Comorbidity						
No	Ref.					
Yes	1.36	0.74–2.50	0.329			
Diabetes						
No	Ref.					
Yes	0.66	0.20–2.23	0.504			
Arterial hypertension						
No	Ref.					
Yes	1.51	0.66–3.45	0.323			
Lung disease						
No	Ref.					
Yes	0.95	0.30–3.04	0.934			
Obesity						
No	Ref.					
Yes	1.58	0.49–5.11	0.446			
Other comorbidities						
No	Ref.					
Yes	1.05	0.53–2.08	0.886			
Clinical scenario						
Mild/moderate	Ref.					
Severe/critical	3.57	1.90–6.72	**<0.001**			
Type of cancer						
Hematological malignancies	Ref.					
Solid tumors	1.52	0.83–2.81	0.173			
Oncological diagnosis						
Acute lymphocytic leukemia	Ref.					
Non-Hodgkin lymphoma	1.40	0.43–4.58	0.582			
^a^Other hematologic malignancies	1.73	0.52–5.77	0.375			
Urological cancer	2.96	0.81–10.88	0.102			
Breast cancer	3.73	1.08–12.91	0.037			
^b^Other solid tumors	1.41	0.47–4.23	0.538			
Cancer stage						
Non-advanced	Ref.					
Advanced	1.61	0.94–2.75	0.082			
Cancer status						
Non-active	Ref.			**Ref.**		
Active	2.96	1.51–5.81	**0.002**	**7.56**	**2.75–20.82**	**<0.001**
C-reactive protein mg/L						
<189.3	Ref.					
≥189.3	1.60	0.77–3.32	0.205			
Leukocytes ×10^9^/L						
<12.8	Ref.			**Ref.**		
≥12.8	2.92	1.45–5.89	**0.003**	**3.00**	**1.17–7.69**	**0.022**
Neutrophils ×10^9^/L						
<7.51	Ref.					
≥7.51	3.55	1.89–6.68	**<0.001**			
Neutrophil/lymphocyte ratio						
<14.5	Ref.					
≥14.5	3.00	1.58–5.65	**0.001**			
Platelets ×10^9^/L						
<393	Ref.					
≥393	1.21	0.50–2.92	0.680			
Fibrinogen g/L						
<9.39	**Ref.**					
≥9.39	**9.94**	**1.17–84.43**	**0.035**			
D-dimer ng/mL						
<1,345	**Ref.**					
≥1,345	**2.57**	**1.38–4.77**	**0.003**			
Lactate dehydrogenase U/L						
<329	**Ref.**					
≥329	**2.54**	**1.35–4.79**	**0.004**			
Urea mmol/L						
<7.4	**Ref.**			**Ref.**		
≥7.4	**7.31**	**3.07–17.43**	**<0.001**	**3.20**	**1.09–9.37**	**0.034**
Creatinine umol/L						
<71	**Ref.**					
≥71	**3.77**	**1.76–8.08**	**0.001**			
Ferritin ng/mL						
<1,640	**Ref.**			**Ref.**		
≥1,640	**3.09**	**1.22–7.83**	**0.017**	**7.22**	**1.79–29.10**	**0.005**
Oxygen saturation %						
<86.9	**Ref.**					
≥86.9	**0.45**	**0.22–0.92**	**0.029**			
Lactate mmol/L						
<2.0	**Ref.**			**Ref.**		
≥2.0	**6.86**	**3.06–15.36**	**<0.001**	**4.79**	**1.79–12.82**	**0.002**
Lung involvement %						
<35	**Ref.**			**Ref.**		
≥35	**3.27**	**1.74–6.15**	**<0.001**	**4.34**	**1.70–11.01**	**0.002**
CO-RADS score						
<5	Ref.					
5	1.61	0.87–2.95	0.126			
Consolidation						
No	Ref.					
Yes	1.52	0.95–2.46	0.081			
Nodular pattern						
No	Ref.					
Yes	1.64	0.89–3.02	0.113			
Frosted glass						
No	Ref.					
Yes	1.56	0.92–2.65	0.097			
Crazy paving						
No	**Ref.**					
Yes	**2.58**	**1.51–4.40**	**0.001**			
Organizing pneumonia						
No	Ref.					
Yes	1.44	0.68–3.04	0.337			
Vessel thickening						
No	**Ref.**					
Yes	**1.91**	**1.16–3.16**	**0.011**			

^a^ Acute myeloid leukemia, chronic myeloid leukemia, mixed phenotype leukemia, multiple myeloma.

^b^ Cervix cancer, head and neck cancer, gastrointestinal cancer, lung cancer, liver and bile duct cancer, thyroid cancer, sarcoma, skin cancer, and other cancer.

Bold format means statistical significance of the *p* value.

## Discussion

The mortality rate in our population during the first wave of the pandemic was higher than that found in cancer patients with COVID-19, whose mortality rates were highly variable [27]. It could be related to underlying clinical conditions. We emphasize that our study was conducted only in hospitalized patients, most of whom had active cancer. In addition, numerous deaths occurred shortly after the diagnosis of COVID-19, which shows delayed medical care resulting from the impact of the pandemic on hospital capacity in our country [28]. The high in-hospital mortality rate was related to the critical baseline condition of the patients.

Oncological characteristics could play a role in the outcome of COVID-19 in our population. The strong association between active cancer stage and an increased risk of death coincides with the findings of some studies [10, 29–32]. Active cancer leads to prothrombotic and proinflammatory conditions that concur with alterations in the immune system due to the development of cancer and antineoplastic treatments [33, 34]. Moreover, SARS-CoV-2 infection results in immune alterations and thrombotic events. Therefore, this could lead to an unfavorable prognosis in patients with cancer and COVID-19 [35, 36]. Advanced or metastatic cancer has been associated with an increased risk of death [37]. In our study, we observed this association, although it was not significant. Findings in this regard are contradictory; some studies have found it to be a risk factor for death in COVID-19 [10, 11, 27, 32], while others have not [12, 38–40]. In contrast, the non-association between the type of cancer, solid tumor or hematological neoplasm, and the probability of death in COVID-19 patients coincides with several studies [10–12, 27, 30–32, 39].

Active cancer represents the main vulnerability of cancer patients in the pandemic, otherwise there is still a need to better understand the possible role of the cancer stage and the type of neoplasia in the outcome of COVID-19.

Mortality in cancer patients with COVID-19 increases with each year of age. Older age leads to a deficiency in the immune response, resulting in a higher risk of death in COVID-19 patients [41]. The strength and direction of the association between age and COVID-19 mortality have been consistently found across multiple studies [10–12, 27, 29–32, 38–40].

The alteration of some laboratory parameters could be a manifestation of the pathophysiology of the disease, but it could also be due to cancer itself.

The high serum concentrations of lactate and, in particular, of ferritin could be the result of a sustained and hyperactive inflammatory response in the COVID-19 patient [3]. Increasing concentrations of serum ferritin, according to the proposed cut-off values, were strongly associated with the mortality of COVID-19 in our population. Serum ferritin levels increase because of cell damage and severe uncontrolled inflammatory conditions [41, 42] including malignant diseases and their progression [43, 44]. The threshold value evaluated (≥1,640 ng/mL) represent a significant increase in serum concentration and correlate with a hyperinflammatory state and a severe clinical course in COVID-19 [45, 46]. Moreover, the increase in serum ferritin has been associated with the severity and mortality of COVID-19 in oncology population, even with lower threshold values [11]. In contrast, a slight increase in serum lactate was associated with mortality. Lactate is a metabolite that is increased in the blood during tissue hypoxia and hypermetabolism, which results in tissue damage and organ failure [47, 48]. Hyperlactatemia (lactate >2.0 mmol/L) is associated with septic shock and mortality in cancer patients with a high incidence of sepsis-related morbidity and mortality [49]. Also, increased values of this analyte in blood, similar to the threshold evaluated in our study (≥2.0 mmol/L), have been found to be a predictor of mortality for COVID-19 in cancer patients, although with a higher threshold value [11]. Serum urea level was another biochemical marker of mortality in the present study. Measurement of uremia is routinely performed to evaluate renal function and its increase as an indicator of renal insufficiency [50, 51]. Although renal insufficiency is a frequent clinical condition in patients with cancer [16, 18], with the consequent increase in serum urea, it is also an important sequela of COVID-19 [52, 53]. The study of blood urea had a good prognostic capacity and association with mortality, although with a slightly higher threshold value (≥7.4 mmol/L). This could indicate the beginning of the manifestation of kidney damage and could also be due to the presence of renal comorbidity in some patients. Increased serum levels of urea (in the form of BUN) have been associated with higher mortality for COVID-19 in cancer patients [54]. Monitoring the evolution of uremia and the study of other complementary tests could contribute to a better understanding of this prognostic factor.

In the hematological study, the leukocyte count seems to be an unspecified marker. An increased leukocyte count represents an inflammatory state resulting from the innate immune response to infection [55, 56]. Likewise, leukocytes are also increased in malignant neoplasms due to the close relationship between the development and progression of cancer and a state of systemic inflammation [57–59]. The increased leukocyte count would indicate a severe inflammatory reaction as a consequence of COVID-19 in cancer patients, even with lower threshold values [12]. However, this finding must be evaluated by considering the oncological context in which many neoplasms frequently maintain leukocytosis, as a subclinical proinflammatory state. In contrast, the total leukocyte count may be affected by the toxicity of antineoplastic therapy in patients under treatment [60, 61]. Contrary to the well-known association found between coagulation markers and COVID-19, we observed no such association. The values of D-dimer, fibrinogen and platelets are frequently altered due to the complication of cancer and its treatment, especially when the disease is active [62, 63].

Although we found some laboratory markers associated with mortality, establishing the prognostic utility of these parameters requires considering that their increased concentration may be due to the underlying neoplasm and/or to the manifestation of the severity of COVID-19.

Chest CT showed a prognostic association with death in COVID-19 through the evaluation of the percentage of affected lungs. To the best of our knowledge there is no association studies between chest CT score system and COVID-19 in oncology patients. However, chest CT can be useful in the study of COVID-19 in cancer patients in which it can show atypical images with few or solitary abnormal findings; therefore, rare or subtle patterns can characterize SARS-CoV-2 infection [13].

CT scoring could help to stratify patient risk and predict short-term outcome in COVID-19 pneumonia [64]. The expression of global pulmonary involvement, regardless of the alteration type, allows us to predict clinical evolution [65], since with the semiquantitative scoring system to estimate lung involvement, all the abnormalities present in the CT are taken into account based on the affected area. Lung involvement score on chest CT in the general population associated with mortality were ≥15 (lung involvement ≥60%) [66, 67] and ≥12.5 [68]. However, in our study, a lung CT score of ≥8.75 (lung involvement ≥35%) was associated with a higher probability of death, which could have been due to limited immune function, especially in patients with hematological neoplasms receiving antineoplastic treatment and with leukopenia [13]. The extent of lung involvement observed on CT, even at a low percentage, could help identify cancer patients at a high risk of death.

Although, there is limited data on chest CT for the diagnosis of COVID-19 pneumonia with a focus on cancer populations [14], in evaluating multiple and non-specific findings, chest CT is a useful tool in the prognosis of death from COVID-19 in cancer patients.

In summary, our clinical study attempted to clarify the role of cancer in the fatality of COVID-19 infection. We found several serum markers and imaging patterns of mortality, but mainly we identified active cancer, cancer progression or its recurrence after treatment, as a critical variable. Our findings derive from a comprehensive clinical, laboratory, and imaging analysis, highlighting the complex interplay of oncologic features and the pathophysiology of COVID-19 to predict the fatal outcome of the disease.

## Limitations

Our study had some limitations that do not invalidate the results; on the contrary, they lead to the proposal of further evaluations to overcome them. First, the small size of the cohort, which probably prohibited to clarify or detect associations due to insufficient statistical power, as we only had access to the complete information of patients with COVID-19 for the study period of time. Second, recognized markers were not evaluated in the COVID-19 study, such as interleukin 6 and procalcitonin, which were not available or were economically unfeasible for developing countries such as ours. Third, neoplastic treatment, which can influence the total leukocyte count, was not analyzed in the study, although the toxic effect of this therapy occurs in all cell types. Finally, cases that had incomplete information could have generated some bias in the results, although the missing data were expected to be undifferentiated for the groups analyzed.

## Conclusions

• Active cancer represents the main prognosis factor of death, while the role of cancer stage and type is unclear.
• Chest CT is a useful tool in the prognosis of death from COVID-19 in cancer patients.
• It is a challenge to establish the prognostic utility of laboratory markers as the alteration of their values can have either oncological or pandemic origins.
• Clinical, laboratory and radiological correlations can help improve the prognosis of death in cancer patients with pulmonary involvement due to COVID-19.

## Data Availability

The raw data supporting the conclusion of this article will be made available by the authors, without undue reservation.
